# Can MOCA Be Applied for Rough Cognitive Assessment in Patients with Epilepsy in Mongolia?

**DOI:** 10.3390/jcm14103372

**Published:** 2025-05-12

**Authors:** Ulziizaya Sodov, Khishigsuren Zuunnast, Hermann Stefan, Tovuudorj Avirmed

**Affiliations:** 1 Intermed Hospital, Ulaanbaatar 17040, Mongolia; ulziizaya.s@intermed.mn; 2 Department of Psychiatry, School of Medicine, Mongolian National University of Medical Sciences, Ulaanbaatar 13270, Mongolia; khishigsuren@mnums.edu.mn; 3 Department of Neurology Biomagnetism, Epilepsy Center, University Hospital Erlangen, 91054 Erlangen, Germany; hermann.stefan@t-online.de; 4 Department of Neurology, School of Medicine, Epilepsy Center, Mongolia-Japan Hospital, Mongolian National University of Medical Sciences, Ulaanbaatar 13270, Mongolia

**Keywords:** cognitive impairment, MoCA, temporal lobe epilepsy

## Abstract

**Introduction:** Epilepsy is a chronic neurological disorder, with cognitive impairment being one of its most significant comorbidities. While the majority of individuals with epilepsy maintain regular intellectual abilities, they are more likely to experience cognitive impairment compared to a healthy control group of the same age and educational level. **Aim:** This study aimed to assess cognitive impairment during epilepsy, particularly temporal lobe epilepsy, and to evaluate the effectiveness of using the Montreal Cognitive Assessment (MoCA) test for cognitive screening in individuals with epilepsy. **Materials and methods:** One hundred and fifty subjects were included between 2022 and 2023, which were divided into 50 people diagnosed with temporal lobe epilepsy (TLE), 50 people with other types of epilepsy according to the International League Against Epilepsy (ILAE), and 50 healthy controls without epilepsy (HC). **Results:** Significant differences were found in the total mean scores of the MoCA between TLE, other types of epilepsy, and healthy control groups (*p* = 0.000), particularly in visuospatial orientation, concentration, memory recall, abstraction, and language skills. **Conclusions:** Evaluating cognitive impairment in epilepsy involves comprehensive neuropsychological assessments, which have significantly advanced in recent years. Nevertheless, we consider the Montreal Cognitive Assessment (MoCA) test to be an appropriate initial screening tool for assessing cognitive impairment in epilepsy.

## 1. Introduction

Epilepsy is one of the most common and widespread neurological disorders, and there are more than 65 million people living with epilepsy in the world [1]. According to the World Health Organization, 80% of all people with epilepsy live in low- and middle-income countries, and approximately 75% of them do not receive appropriate treatment. An average of 5000 people are diagnosed with epilepsy per year, with an annual incidence of 49 per 100,000 in developed countries and 139 per 100,000 in low- and middle-income countries (WHO). In a systematic review and meta-analysis, the incidence of epilepsy was 61.4 (95% CI 50.7–74.4) per 100,000 person-years [2]. In our country, a study by Y. Erdenechimeg et al. (2005) reported that the prevalence of epilepsy among the population aged five and above in Ulaanbaatar is 2.5 per 1000 individuals [3]. Additionally, according to the Health Indicators 2019 statistical report of Mongolia, epilepsy has accounted for an average of 10.2% of neurological disorders over the past decade [4].

Recently, several studies have shown that recurrent seizures affect all aspects of cognitive functioning, including attention, language, praxis, executive function (intelligence), judgment, insight, and problem-solving [5]. Long-term epilepsy research has shown that uncontrolled, chronic, severe seizures cause memory loss in 17–38% of individuals with episodes lasting 2–10 years [6]. The left hemisphere of the brain plays a major role in memory function, and a comparative study of the right and left hemispheres in patients with epilepsy and hippocampal sclerosis showed that the left hemisphere had a lower score on the verbal memory test [7]. In Mongolia, research on cognitive impairment during epilepsy, especially temporal lobe epilepsy, is exceptionally rare. In 2018, Orkhonselenge et al. studied 170 individuals with epilepsy and found that cognitive decline affected medication adherence. The MoCA test was used in this study, with an average score of 17.57 ± 0.64 [8].

There are various cognitive screening tools available (e.g., MMSE, MoCA, EpiTrack, Neuropsychiatry Unit Cognitive Assessment Tool, MiniCog). However, it is crucial to consider metrics that are compatible across languages and cultures when evaluating cognitive decline in epilepsy, especially in low–middle-income countries [9]. The MoCA is a highly promising cognitive screening tool, translated into nearly 100 languages and supported by comprehensive training and quality control procedures. It is increasingly used worldwide across various health conditions. The MoCA boasts high specificity (87%) and sensitivity (90%) for detecting mild cognitive impairment, and it assesses executive function, language abilities, and visuospatial processing more rigorously than the Mini-Mental State Examination (MMSE) [10]. Additionally, the MoCA test has proven to be an effective screening tool for identifying cognitive impairments in epilepsy patients, with an overall accuracy of 72%, sensitivity of 88.2%, and specificity of 63.6% in outpatient settings [11,12,13]. In our country, the MoCA test is being used for the first time in epilepsy care, and evaluating cognitive impairment with this test is essential for planning treatment, improving care, preventing neuropsychiatric complications, reducing work disability, and facilitating occupational adjustments.

## 2. Material and Methodology

One hundred and fifty subjects were included between 2022 and 2023, which were divided into 50 people diagnosed with temporal lobe epilepsy (TLE), 50 people with other types of epilepsy according to the International League Against Epilepsy (ILAE), and 50 healthy controls without epilepsy (HC). The data were obtained through anamnesis performed on patients assessed at the National Center for Mental Health and the Epilepsy Center of the Mongolia-Japan Hospital of MNUMS. The MoCA test was conducted by a neurologist, and for individuals with less than 12 years of education, 1 point was added to their score. We excluded patients who refused participation, had a structural abnormality in magnetic resonance imaging (MRI), progressive neuropathological conditions, and conditions that significantly interfere with cognitive functions (an intellectual disability, diabetes, liver disease, and a primary or uncontrolled psychiatric disorder).

### 2.1. Instruments

The Mongolian translated paper version of the MoCA (in 1996 by Ziad Nasreddine in Montreal, QC, Canada) test was used and classified as follows: mild (18–25), moderate (10–17), severe (below 10), and normal (above 26).

### 2.2. Statistical Analyses

A statistical distribution and normality analysis was performed. Subsequently, Student’s t-test and the Chi-square test with an alpha level of 0.05 were conducted to verify the differences in MoCA cognitive performances between groups. In our study, cognitive impairment was considered a dependent variable, while age, gender, education, and seizure-related features were considered independent variables. In order to analyze MoCA score differences between the three groups, a one-way ANOVA test was used. Finally, logistic regression analysis was used to identify potential risk factors affecting cognitive impairment.

All statistical analyses were performed using STATA program V.17.0 (2021, April, College Station, TX, USA) and IBM SPSS V.28.0 (2021, May, Chicago, IL, USA).

### 2.3. Ethical Aspects

Upon initiating our research, we adhered to the principles outlined in the Declaration of Helsinki (1975, revised in 2013) [14]. All participants, including cases and controls, were informed about the study and provided their informed consent. The research was approved by the ethics committee board of the Mongolian National University of Medical Sciences (Protocol No. 3-01, 1 January 2022).

## 3. Results

In our study, we included 150 participants aged 18–63 years, with 77 males (51.3%) and 73 females (48.7%). The socio-demographic characteristics of the study participants are presented in Table 1.

The mean age was 42.5 ± 10.07 years; the age at seizure onset ranged from 1 to 50 years, with a mean onset age of 17.14 ± 9.21 years. The epilepsy group exhibited relatively lower levels of education and socioeconomic status compared to the control group, which were significantly different. The clinical characteristics of epilepsy are shown in Table 2. Most patients experienced complex partial seizures and were on antiseizure medication monotherapy. Notably, 82% had been using antiseizure medication for over 10 years, with Carbamazepine being the most commonly used medication.

Table 3 describes the cognition impairment experienced by subjects between groups (TLE, other types of epilepsy, and HC). The temporal lobe epilepsy group showed a significantly higher level of moderate cognitive impairment.

The mean MoCA score was 17.5 ± 4.5 for the temporal lobe epilepsy group, 20.0 ± 4.4 for the other types of epilepsy group, and 26.4 ± 2.3 for the healthy control group. The relatively lower scores in the temporal lobe epilepsy group were significantly different (95% CI 16.19–18.80, *p* = 0.000). Using logistic regression analysis, it was found that age, a low educational level, higher seizure frequency, and duration of epilepsy are significant risk factors for cognitive impairment, while a lower seizure frequency acts as a protective factor (Table 4).

Table 5 describes the performance of subjects across the three groups in the cognitive test. To analyze the statistical differences in numerical values among these groups, a one-way ANOVA test with Bonferroni correction was employed.

Both epilepsy groups (other types of epilepsy and TLE) have significantly lower MoCA scores compared to healthy controls. The TLE group has significantly lower MoCA scores compared to the other types of epilepsy group, indicating more severe cognitive impairment in the TLE group. Upon reviewing the results in the subscales of the instrument, the epilepsy group had a poorer performance in visuospatial orientation, concentration, memory recall, abstraction, and language skills. In TLE, visuospatial orientation, naming, concentration, and language skills were more significantly impaired compared to the other types of epilepsy group.

## 4. Discussion

Cognitive impairment is prevalent in epilepsy; however, epilepsy care often prioritizes seizure management over cognitive assessment and intervention. Addressing cognitive decline is crucial for comprehensive epilepsy care and improving patient outcomes. Therefore, this study aimed to evaluate cognition impairment using the MoCA test.

The group with epilepsy had lower average scores than healthy controls in the MoCA test, indicating significant overall cognitive impairment. These results are consistent with the other relative studies on cognition impairment and using the MoCA test [11,15,16]. Phabphal K et al. reported that cognitive decline can be detected using the Montreal Cognitive Assessment (MoCA) even when the Mini-Mental State Examination (MMSE) scores are normal in epilepsy patients and recommended using the MoCA test for cognitive assessment in epilepsy [17]. In a study comparing MoCA and MMSE in epilepsy patients [18], it was found that MOCA is more sensitive than MMSE in demonstrating cognitive impairment in epilepsy. In our study, 68% of participants exhibited cognitive decline, with noticeable impairments in language skills, concentration, and memory. These findings are consistent with numerous studies on cognitive decline in epilepsy [19,20,21].

The MoCA test employs tasks such as clock drawing to evaluate visuospatial orientation and abstract thinking, which are particularly significant. Our study demonstrated that visuospatial orientation was more significantly impaired in the group with temporal lobe epilepsy (TLE) compared to other epilepsy groups. This finding underscores the MoCA test’s capability to provide a detailed assessment of various cognitive domains, highlighting its utility in detecting specific cognitive impairments in epilepsy patients. Attention, memory, abstraction, and language impairments were identified in patients with prolonged epilepsy duration and increased seizure frequency using the MoCA test [22].

The early detection and monitoring of cognitive decline in epilepsy are crucial [23]; however, routine cognitive assessments are relatively limited in low- and middle-income countries. Additionally, the high prevalence of cognitive decline among patients with epilepsy suggests that undergoing neuropsychological evaluations after an epilepsy diagnosis is advisable, as these assessments can inform treatment plans and serve as initial indicators for rehabilitative care [24]. Therefore, the implementation of brief and simple cognitive tests in clinical practice, particularly in outpatient settings, is essential for the early detection and monitoring of cognitive decline in epilepsy. The Montreal Cognitive Assessment (MoCA) test is deemed suitable for addressing this need.

### Limitations of the Study

Our study encountered several limitations, including a small sample size, an older average age in the study groups, a prolonged duration of epilepsy, limited access to treatment, and a narrow selection of antiepileptic drugs. These factors likely influenced the study outcomes and contributed to the lower average scores on the Montreal Cognitive Assessment. Additionally, the low educational level of participants emerged as a risk factor for cognitive decline. Furthermore, our study did not thoroughly compare memory loss with cognitive impairment, which is another limitation. For future research, we aim to increase the sample size, utilize more sensitive tests to assess cognitive impairment before and after temporal lobe surgery, compare multiple tests, and include assessments of memory loss. By addressing these limitations, we hope to gain a more comprehensive understanding of cognitive impairment in epilepsy patients and improve the effectiveness of interventions.

## 5. Conclusions

Evaluating cognitive impairment in epilepsy involves comprehensive neuropsychological assessments, which have significantly advanced in recent years. Nevertheless, we consider the Montreal Cognitive Assessment (MoCA) test to be an appropriate initial screening tool for assessing cognitive impairment in epilepsy. We believe that its implementation will greatly enhance the quality of care provided to epilepsy patients.

## Figures and Tables

**Table 1:** General socio-demographic characteristics of the study participants.

Variables	TLE *n* (%)	Other Epilepsy *n* (%)	HC *n* (%)	*p* Value
Mean age	43.78 ± 8.20	41.46 ± 11.38	42.26 ± 10.43	0.41
Seizure onset age	15.02 ± 9.28	19.26 ± 8.73	/-/ *	0.01
Average duration of living with disability	20.18 ± 6.88	16.56 ± 9.07	/-/	0.01
Gender				
Male	26 (52%)	26 (52%)	25 (50%)	
Female	24 (48%)	24 (48%)	25 (50%)	
Education level				
Low or no education	11 (22%)	12 (24%)	4 (8%)	0.01
Secondary or vocational	33 (66%)	26 (52%)	24 (48%)	0.24
High education	6 (12%)	12 (24%)	22 (44%)	0.001
Marital status				
Married	13 (26%)	22 (44%)	31 (62%)	
Single	37 (54%)	28 (56%)	19 (38%)	0.002
Socioeconomic status				
With disability	46 (92%)	40 (80%)	/-/	
Employed	1 (2%)	6 (12%)	48 (96%)	
Unemplyoed	3 (6%)	4 (8%)	2 (4%)	

* In the healthy control group, there is no data available for seizures and disabilities.

**Table 2:** Seizure characteristics.

Variables	TLE *n* (%)	Other Epilepsy *n* (%)	*p* Value
Seizure onset age (mean)	15.02 ± 9.28	19.26 ± 8.73	0.01
Seizure duration years (mean)	28.76 ± 8.79	22.20 ± 11.36	0.000
Living with disability years (mean)	20.18 ± 6.88	16.56 ± 9.07	0.01
Seizure types			
Complex partial seizures	37 (74%)	18 (36%)	0.000
Simple partial seizures	13 (26%)	32 (64%)	
Seizure onset age			
Above 14 years old	21 (42%)	38 (76%)	
Below 14 years old	29 (58%)	12 (24%)	0.001
Seizure duration years			
Above 16 years	38 (76%)	19 (38%)	0.000
Below 16 years	12 (24%)	31 (62%)	
Seizure frequency			
Constant	28 (56%)	17 (34%)	0.02
Rare	9 (18%)	21 (42%)	
Epileptic status			
Yes	15 (30%)	9 (18%)	0.160
No	35 (70%)	41 (82%)	
Presence of aura			
Yes	28 (56%)	18 (36%)	0.04
No	22 (44%)	32 (64%)	
Post-ictal automatism			
Yes	44 (88%)	18 (36%)	0.000
No	6 (12%)	32 (64%)	

**Table 3:** Cognitive impairment status.

Cognition Impairment	TLE *n* (%)	Other Epilepsy *n* (%)	HC *n* (%)	*p* Value
Normal	4 (8%)	8 (16%)	36 (72%)	0.000
Mild cognitive impairmen	17 (34%)	24 (48%)	14 (28%)	0.62
Moderate cognitive impairment	29 (58%)	18 (36%)	0 (0%)	0.000

**Table 4:** Logistic regression analysis of risk factors for cognitive impairment.

Independent Variables	OR	95% CI	*p* Value
Age (years)	1.14	1.05–1.22	0.001
Education level (low)	3.77	1.04–13.6	0.04
Seizure frequency (high)	9.5	2.37–38.6	0.002
Seizure duration (years)	1.14	1.05–1.22	0.000

**Table 5:** Statistical differences in cognitive test performance (ANOVA).

MoCA Test Performance	TLE Score (SD)	Other Types of Epilepsy Score (SD)	HC Score (SD)	Mean Difference Between Groups *	*p* Value
MoCA score	17.5 (4.5)	19.9 (4.4)	26.46 (2.3)	A: −6.46	0.000
				B: −8.96	0.000
				C: −2.50	0.005
Visiuspatial	2.28 (1.61)	3.08 (1.2)	4.48 (0.6)	A: −1.4	0.000
				B: −2.2	0.000
				C: −0.8	0.004
Naming	2.5 (0.70)	2.7 (0.43)	2.9 (0.2)	A: −0.16	0.343
				B: −0.42	0.000
				C: −0.26	0.033
Concentration	3.04 (1.53)	3.82 (1.58)	5.2 (0.9)	A: −1.22	0.000
				B: −2.18	0.000
				C: −0.96	0.001
Recall memory	1.02 (0.89)	1.12 (1.15)	3.06 (1.2)	A: −1.94	0.000
				B: −2.04	0.000
				C: −0.1	1.00
Language	1.52 (0.81)	1.84 (0.73)	2.6 (0.6)	A: −0.78	0.000
				B: −1.1	0.000
				C: −0.32	0.09
Abstraction	0.94 (0.73)	1.1 (0.78)	1.8 (0.3)	A: −0.74	0.000
				B: −0.9	0.000
				C: −0.16	0.682
Orientation	5.48 (0.83)	5.28 (0.88)	5.8 (0.3)	A: −0.56	0.001
				B: −0.36	0.04
				C: 0.2	0.526

* Comparison A: HC vs. other types of epilepsy; B: HC vs. TLE; C: other types of epilepsy vs. TLE.

## Data Availability

The data presented in this study are available upon request from the corresponding author. The data are not publicly available due to specific ethical and privacy considerations.
